# Mitochondrion: A Promising Target for Nanoparticle-Based Vaccine Delivery Systems

**DOI:** 10.3390/vaccines4020018

**Published:** 2016-06-01

**Authors:** Ru Wen, Afoma C. Umeano, Lily Francis, Nivita Sharma, Smanla Tundup, Shanta Dhar

**Affiliations:** 1 NanoTherapeutics Research Laboratory, Department of Chemistry, University of Georgia, Athens, GA 30602, USA; soso2012@uga.edu (R.W.); aumeano@uga.edu (A.C.U.); lgf39148@uga.edu (L.F.); nivita.sharma97@uga.edu (N.S.); 2 School of Medicine, Department of Pulmonary and Critical Care, University of Virginia, Charlottesville, WV 22908, USA; st2vk@virginia.edu

**Keywords:** vaccine, mitochondria targeting, nanoparticles, antigen delivery

## Abstract

Vaccination is one of the most popular technologies in disease prevention and eradication. It is promising to improve immunization efficiency by using vectors and/or adjuvant delivery systems. Nanoparticle (NP)-based delivery systems have attracted increasing interest due to enhancement of antigen uptake *via* prevention of vaccine degradation in the biological environment and the intrinsic immune-stimulatory properties of the materials. Mitochondria play paramount roles in cell life and death and are promising targets for vaccine delivery systems to effectively induce immune responses. In this review, we focus on NPs-based delivery systems with surfaces that can be manipulated by using mitochondria targeting moieties for intervention in health and disease.

## 1. Introduction

**Figure 1:**
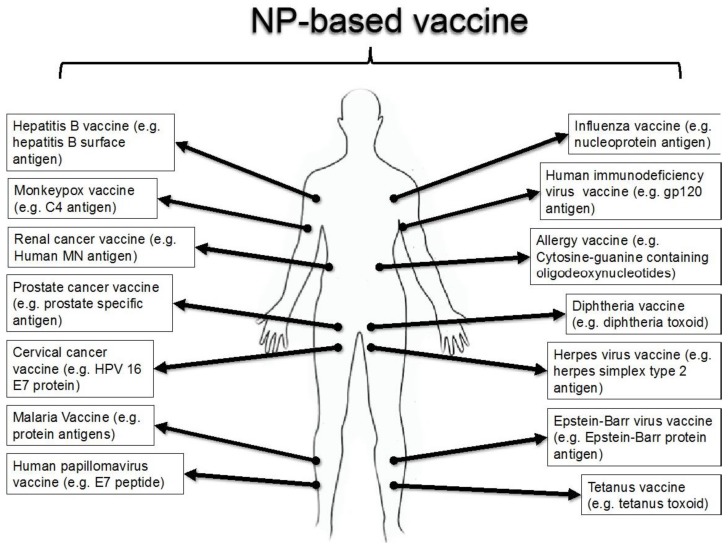
Overview of possible vaccines that can be generated using NPs.

Vaccines are designed immunogenic antigens used to intentionally trigger the memory component of the immune system by stimulating humoral immunity *via* the production of antibodies for long term protection against various diseases [1] (Figure 1). Attenuated or inactivated vaccine can elicit immunoprotection but duration of effect is limited and concerted to cellular immune responses [2]. Vectors and/or adjuvant delivery systems are widely used to augment immunogenicity of antigens, to protect vaccines from degradation in physiological environment, to improve efficacy of vaccines, and to target specific sites preventing unwanted accumulation [3].

For effective therapeutic use, vectors should be stable, biodegradable, biocompatible, easy to prepare, low cost, immunologically inert, and/or serve synergistically as an adjuvant. Nanoparticle (NP)-based payload cargos and adjuvants for vaccines are growing technologies due to their intrinsic immune-stimulatory properties, ability to co-entrap antigen adjuvants such as toll-like receptor (TLR) and enhancement of the antigen uptake by cells, e.g., by professional antigen presenting cell (APC) manipulation [4,5,6].

Mitochondria play essential roles for life and death processes of cells. This complex organelle participate in energy generation, intermediary metabolism, exchange of information, calcium signaling, and regulation of apoptosis [7,8,9]. Mitochondria-associated dysfunctions provide a predictable prospectus of defects in tissues for many ailments, spanning from subtle alterations causing symptomatic illness, to major functional defects leading to death [10,11]. Mitochondria play an important role in the immune system, which involves signaling platforms, effector responses [12], and modulating the antigen-specific T cell activation *via* reactive oxygen species (ROS) signaling [13,14]. Mitochondrial antigens, such as M2 autoantigens [15], oxo-acid dehydrogenase complexes [16] and 2-oxoglutarate dehydrogenase complex [17], are known to induce disease-related autoimmune responses such as primary biliary cirrhosis (PBC) [15,16,17,18,19]. The targets located in the different compartments of mitochondria for possible vaccine development are listed in Table 1. There are reports of mitochondria-targeted NP systems with the ability to produce tumor associated antigens (TAAs) that have the potential to act as preventive/therapeutic vaccines [20]. A mitochondria targeted vaccine was recently reported to stimulate the immune response by mitochondrial DNA (mtDNA) mutations upon immunization in a renal cancer murine model [21]. Such findings support previous studies performed by West *et al.* illustrating that mtDNA stress primes immune response [22], which may be critical to encourage effective immune stimulation, modulation, and memory-like protection in the context of vaccines. Therefore, mitochondria are promising targets for vaccine delivery systems to effectively induce immunity against diseases and to establish and/or boost therapeutic effects.

Although mitochondria can serve as important modulators for vaccines by serving as unique targets, the field of mitochondria targeted vaccines by using NP delivery system is still in infancy. This might be due to limited availability of NP systems for vaccine design. In this review, we highlight recent advances of antigen delivery carriers that can be manipulated to achieve mitochondria targeting and their potential interventions as preventive/therapeutic vaccines, by categorizing them into compositional classes of: (a) polymeric; (b) liposomal; and (c) other types of antigen carrier systems. Along with discussion of mitochondria targeting moieties, examples of mitochondria targeted NP vaccines are provided as well as future directions for this field.

## 2. NP-based Vaccine Delivery Systems

NP-based therapeutics are emerging as a noteworthy field in clinical research. Currently, there are several FDA approved cancer nanomedicines, such as Abraxane, Doxil, Oncaspar, *etc.* NPs provide an important tool for clinical application and, thus, should and can be evolved for systems that aim for various functions, such as in the case of vaccines. Adjuvant or antigen-targeting delivery systems are critical for efficient immune modulation by protecting antigens from extracellular enzymes and pH changes, and translocation of antigens to the target sites [32]. Here, we have categorized these NP-based vaccine delivery systems as polymeric, liposome, and others types of NP based carriers.

### 2.1. Polymeric NPs as Antigen Carriers

Antigens loaded into nanometer-sized polymeric particles were recently shown to produce good adjuvant effect [33,34,35,36]. Polymeric NPs are used as adjuvants or antigen delivery cargos that can potentially be used in mitochondria-targeted vaccines. Polymeric NP are desirable platforms due to relative ease in tailoring (i) biodegradation properties and (ii) physical properties, such as size, surface charge, and hydrophobicity, for optimum cargo delivery/circulation and mitochondrial access. A list of polymeric NP based antigen delivery systems is summarized in Table 2.

Biodegradable NPs constructed from poly(lactic-co-glycolic acid) (PLGA) polymer are widely used as antigen carriers/adjuvants due to its biocompatible and biodegradable characteristics, with a safety profile approved by the US Food and Drug Administration (FDA) and European Medicine Agency (EMA) [37], good and controlled long-term release which results in strong immune response. Furthermore, PLGA NPs can carry a variety of antigens including gp120 and gp140 of the human immunodeficiency virus [38,39,40]. Not only were PLGA adjuvant NP systems reported to elicit a strong T cell immune response using 100-fold lower doses (0.05 µg) of CpG oligodeoxynucleotide antigen, NP systems also showed significantly higher cytokine secretion (up to 10-fold), as well as a comparative antibody response to normal saline delivery [41].Thus, PLGA systems may be further developed for tailored vaccines towards increasing Th1 cell and innate immune response (e.g., viral hepatitis clearance).

There are considerable challenges that must be overcome in order to develop strong PLGA-based NP systems for vaccines. For one, PLGA-based NPs have poor payload loading and display accelerated burst release of vaccines to unwanted tissue or cells. Thus, several modifications have been done to overcome these issues. For examples, a pH sensitive PLGA NPs system was developed for rapid release of ovalbumin (OVA) antigen in acidic environments to improve immune response [42]. NPs were prepared by combination methods of emulsion-diffusion-extraction and emulsification and were ~890 nm in diameter with zeta potentials of ~ −12 mV. The antigen release profile was pH-dependent with over 85% release in acidic environments (pH 5.0 or 6.5) and low release (10%) at physiological pH for 24 h. *In* *vitro* studies showed that PLGA-OVA NPs significantly enhanced CD86 and CD40 co-stimulatory factors, which induced higher level of cytokines IL-1*β*, IL-6, IL-12p70 and TNF-α than their control groups. *In* *vivo* studies of PLGA-OVA NPs demonstrated enhanced activation of B cells, CD8^+^ T cells, IgG titers, and Th1 polarization than their non-pH responsive control.

Delivery of dual or triple antigens in PLGA-NPs is also a possible strategy to enhance immune response efficiency. Co-delivery of TLR 4 ligand and TAA using PLGA-based NPs were reported to stimulate strong anti-tumor immune response [43]. This dual antigen loaded PLGA-based NP was prepared by double emulsion/solvent evaporation technique and vaccination of these NPs in healthy mice activated CD8^+^ T cell immune responses with greater Th1 cytokines including IFN-*γ*, TNF-α, and IL-2, -6, -12 production in both lymph nodes and spleen compared to 7-acyl lipid A NPs and empty-NP immunization. Polyethylene glycol (PEG) is a flexible, biocompatible, inert, amphiphilic, and non-toxic polymer that is FDA-approved for use in human. PEG is water soluble and, in NPs, prevents interactions between NPs and the cell surface environment. However, PEG-NP’s hydrophilic properties may lead to poor recognition and cellular uptake. NPs from other polymers such as poly(d,l-lactide) (PLA), poly (anhydride), poly(methyl methacrylate) (PMMA), poly(glycolic acid) (PGA), poly(*ε*-caprolactone) (PCL), chitosan, poly-L-lysine, poly(*γ*-glutamic acid) (PGA), can also serve as carriers for vaccine delivery.

Conjugation of two or more types of polymers by creating block copolymers, such as PLGA-*b*-PEG and PLA-*b*-PEG, are of particular interests as NPs carriers in vaccine development. Copolymers can be varied for ratios that to allow for the combined advantages of individual polymers and maximizes delivery and uptake efficiency. Factors including chain length of polymers, particle size, targeting moiety, and surface properties of NPs play significant roles in cellular uptake and immune efficacy of antigen. For example, Cruz *et al.* demonstrated significant effects of chain length of PEG and targeting moiety of antibody on the vaccine delivery and immune responses [44]. Chemically modified PLGA NPs were prepared by emulsification/solvent diffusion with various chain lengths of PEG *via* coupling to activated carboxyl groups. The NPs surface was coated with an antibody (hD1 or ZN-D1) identifying receptor for dendritic cells (DCs). The size of modified PLGA NPs increased with PEG chain length. The PLGA modified NPs with shorter chain length of PEG (MW: 2000–3000) and hD1 antibody showed higher uptake efficiency than longer PEG chain (MW: 6000–20000) modified larger NPs either with or without hD1 antibody. PLGA modified NPs coated with hD1 or AZN-D1 were more efficient for cellular uptake and targeting than those coated with H200, neck motif receptor. These results indicated that chain length of PEG and antibody types influence the ligand-receptor targeting by tailoring the size and surface properties of NPs. Plasmid DNA was loaded into PLGA-polyethyleneimine (PEI) NPs with positive surface charge of 40–70 mV and size of 230–280 nm by precipitation-evaporation-filtration method [45]. *In vitro* studies showed that PLGA-PEI-DNA NPs could stimulate human DCs to secrete IL-12 and TNF-α. *In vivo* study demonstrated enhanced T cell proliferation by immunization with PLGA-PEI-DNA NPs.

Biopolymers, like chitosan, can also be used to prepare comparably cost-effective NPs as stable carriers for antigens to trigger immune response for diseases, such as Hepatitis B, Tetanus, and Leishamaniasis [46,47,48]. For example, chitosan NPs prepared by ionic gelation were developed to successfully deliver TLR3 agonist poly (I:C) (pIC) and a T-Helper peptide (PADRE) to produce antibody against disease [49]. *In vivo* studies in mice indicated that both immunostimulant pIC and T helper peptide were critical to reach valuable immune responses upon immunization with chitosan NPs. The present condition of antigen in the NPs was an important factor for immune responses, whereas the adsorption of peptide on the surface of NPs showed higher antibody response than the entrapping counterpart.

### 2.2. Liposomal Systems as Antigen Carrier/Adjuvant

Liposomes are effective antigen carriers due to biodegradable, nontoxic, non-immunogenic, and antigen loading properties. These systems are reported to deliver a wide range of antigens, such as E7 peptide and HSP70, which can potentially vaccinate against many disease, such as cancer and human papilloma virus [14,60]. Table 3 summarizes the liposome-based NPs in vaccine delivery systems. Lipids, such as dioleoyl phosphatidyl ethanolamine (DOPE), dioleoyl phosphatidylcholine (DOPC) cholesterol, dioleoyldimethylammonium chloride (DODAC), dioleoyldimethylaminopropane (DOTAP), dipalmitoylphospatidylcholine (DPPC), dipalmitoylphospatidylglycerol (DPPG), can be used to prepare liposome-based NP delivery systems for vaccines. The endosomolytic lipid DOPE can enhance the efficacy of vaccine delivery by endosomal escape through membrane disruption and fusion processes [61]. For instance, Cui *et al.* [62] reported DOPE as lipid source for preparation of liposome-based adjuvant NPs as plasmid DNA (pDNA) vaccine carriers. The pDNA-lipid adjuvant NPs had a 250-fold enhanced activity than pDNA alone for *in vivo* immunization in BALB/c mice model.

Liposomal NPs are potentially able to cross epithelial barriers and protect encapsulated antigens from enzymatic attack in physiological environment [63]. Oral route delivery of vaccine is one of the most challenging and difficult tasks in vaccine development. Towards the development of oral or nasal vaccines, liposomal NPs can undergo reverse denaturation, surviving GI tract and other enzyme-rich environments. However, liposomal NPs are unstable and rapidly cleared by the mononuclear phagocyte system (MPS). The combination of liposome and polymer is a possible strategy for construction of antigen nanovesicles to resolve deficiencies in uptake efficacy and immune response enhancement. Many types of combination nanocarriers, such as liposome-chitosan NPs [64], were reported for drug-delivery and such systems may be further evolved for antigen delivery as well. Polymerized liposomes, which can be prepared under mild conditions comparable to conventional liposomes, exhibit greater encapsulation efficiency, better activity of antigen, and more controllable antigen release profile than conventional liposomes. The solid and stable structure of polymerized liposomes with easy preparation method make these NPs a promising antigen delivery vesicle [65]. Polymerized liposomes showed their potential as oral vaccine delivery system [66], however, further studies are required for clinical application.

Solid Lipid NPs (SLNPs) with a diameter of 50–1000 nm can be prepared by replacing the liquid lipid by various types of solid lipids, such as fatty acids, triglycerides, and their combinations [67]. The uptake efficiency and immunogenicity of antigen loaded SLNPs can be affected by size, lipid source, preparation method, and surface properties. For example, Bhargava *et al.* reported SLNPs as tumor antigen carriers, which demonstrated immune signal induction with no toxicity [68]. With tumor lysate as antigen, SLNPs were synthesized by solvent injection method with tristearin as a lipid source. SLNPs size was controlled by varying preparation parameters. The monomannosyl–dioleyl glycerol modified SLNPs showed the highest uptake efficiency and immune response among other lipid modified controls, which may be attributed to efficient antigen capture by mannose receptors of DCs.

The efficacious delivery and safety profile of liposome-based NP systems make them as promising vehicles for vaccine delivery. The immune-stimulant sipuleucel-T received FDA approval for asymptomatic or minimally symptomatic prostate cancer in 2010 and is used to prime metastatic prostate cancer patient’s T-cells to target the patient’s own cancer cells. Liposomes formulated prostate-specific antigen (PSA) is reported to generate immune response when injected into patients (65–80 years old) with prostate cancer. Out of 10, 8 patients showed PSA-reactive T-cell frequencies using *in vitro* sensitization method. The vaccination trial indicated that the patients survived within 22–33 months with one still alive during the testing timeline, whereas eight patients were stable and the rest of them deteriorated to progressive disease [69]. Another pilot study demonstrated the safety profile of MUC1 BLP25 liposomal vaccine. L-BLP25 was prepared by encapsulating BLP25 lipopeptide to form liposomal adjuvant with monophosphoryl lipid A and 3 lipids (cholesterol, dimyristoyl phosphatidylglycerol and dipalmitoyl phosphatidylcholine). Twenty patients (≥18-year-old) were continuously vaccinated with BLP25 liposome vaccine after single dose of cyclophosphamide for up to 1 year. No autoimmunity was observed, indicating that L-BLP25 vaccine is safe for therapeutic use [70]. A recent internationally randomized and double-blind phase III clinical trial composed of 1513 patients with non-small-cell unresectable stage III lung cancer, tested L-BLP25 as an immunotherapeutic [71]. The overall survival was 25.6 months for patients vaccinated with L-BLP25 compared to 22.3 months for patients treated with placebo. These results indicate the potential clinical benefit of L-BLP25 for cancer immunotherapy and warrant further studies [71].

### 2.3. Others Types of NP-based Systems as Antigen Carrier/Adjuvant

There are reports on other NP carriers for the delivery of vaccines other than polymeric or liposome-based NPs. For instance, inorganic NPs are suitable antigen delivery systems due to wide availability, rich functionality, and good biocompatibility. Many inorganic materials, such as aluminum salt, silver, gold, carbon materials, silicon oxide, and iron oxide were studied for use as antigen delivery carriers. Table 4 summarizes these NP carriers for antigen delivery. For example, Villa *et al.* [72] reported single-wall carbon nanotubes (SWNTs) as human tumor antigens carriers to improve immune responses. SWNT-peptide delivery systems were constructed using weakly immunogenic cancer-associated peptide WT1-Pep427 consisting of 19 amino acids. The SWNT-peptide delivery systems showed enhanced *in vivo* immune responses in mice model. More importantly, SWNTs alone did not show any toxicity or immunogenicity *in vitro* or *in vivo*, and no immunogenic responses were reported for this SWNT construct.

Layered double hydroxides NPs (LDH-NPs) are considered as potential vaccine nanocarriers due to good biocompatibility, low toxicity, great antigen protective and controllable release properties. LDH-NPs are hydrotalcite-like anionic clays with a common formula as [M^2+^_1−x_M^3+^_x_(OH)_2_]^x+^(A^n−^)_x/n_·yH_2_O, where M is a metal cation (e.g., Mg^2+^, Al^3+^) located at octahedral sites and A is a inorganic anion (e.g., Cl^−^, CO_3_^2−^) situated at interlayers [73]. LDH-NPs provides efficient uptake of antigens due to their high endosomal buffering capacity and controllable released profiles [74]. Yan *et al.* demonstrated that OVA loaded LDH-NPs (~100 nm in diameter) prepared by a combination of precipitation and hydrothermal methods exhibited less inflammation but comparable adjuvanticity to induce Th1/Th2 immune response [75]. Modification of OVA-LDH-NPs by CpG receptor ligand significantly enhanced immune response. *In vivo* studies showed that LDH-CpG-OVA NP injections had six-fold of IgG2a/IgG1 ratio and less inflammatory activity than mice treated with Alum-CpG-OVA. Hybrid of different types of NPs to combine the advantages of individual NPs is an effective strategy to enhance vaccine immune response. This hybrid strategy is widely used in drug delivery, and can also be applied to vaccine developments. For example Wang *et al.* reported SiO_2_@LDH NPs as adjuvant for DNA vaccination to improve immune response for hepatitis B [76]. Core-shell SiO_2_@LDH (~210 nm in diameter) NPs showed adjuvant activity for maturation of DC *in vitro* and enhanced immune responses with greater generation of IFN-*γ*, IL-6, MHCII, and CD86 *in vivo*. However, toxicity of LDH NPs needs to be addressed for clinical applications.

## 3. Mitochondria Targeting Moiety

As mentioned previously, mitochondria have unique immunostimulatory capabilities that can enhance vaccine activity. Mitochondria targeting can be achieved by selectively conjugating the polymer or modifying the particle surface with a mitochondria targeting moiety. The unique properties of the mitochondrion, such as the existence of a mitochondrial membrane potential (Δ*ψ*_m_) across a mitochondrion’s double membrane, and the mitochondrial protein import machinery indicates that lipophilic cations and specific mitochondrial targeting sequences (MTS) can be used to achieve effective mitochondria targeting.

(i) Lipophilic cation: One approach to confer mitochondria targeting properties to a vaccine is through conjugation to a delocalized lipophilic cation, such as triphenylphosphonium (TPP) cation [28,56,117], rhodamine 123 [118], or methyltriphenylphosphonium (TPMP) cation [119]. Lipophilic cations access the mitochondrion through a driving force caused by the Δ*ψ*_m_ gradient [120]. Cancer cells display a hyperpolarized Δ*ψ*_m_ across membranes in their mitochondrial population, which facilitate the accumulation of lipophilic cations. Thus, lipophilic cation conjugated NPs can be used as carriers to target the antigens to mitochondria. A summary of mitochondria targeting moieties is shown in Table 5.

TPP is a commonly used cation for mitochondria targeting due to its characteristic facile conjugation and efficient rapid uptake into mitochondrion. Nevertheless, the toxicity of TPP-based small organic molecules limits its use in therapeutic applications. Conjugation of TPP cations into a stable polymer, lipid or other nanomaterials can solve this problem. TPP derived polymers used as mitochondria targeting moiety for polymer surface manipulation as delivery systems exhibited less toxicity in recent studies. We recently reported a biocompatible polymeric NP for mitochondria targeting, where biodegradable PLGA-*b*-PEG was functionalized with a terminal TPP cation, forming PLGA-PEG-TPP [56,131]. This polymer has the advantages of being non-toxic, easy to prepare, and stable. Furthermore, it demonstrated success in efficient mitochondria associated delivery of different small molecules which included Pt(IV) compounds [132,133,134], ZnPc [21,27], aspirin [135]. Tagging to other nanomaterials with TPP cation is widely used and achieved by our group. For instance, gold nanoparticle (AuNP) to 3-bromopyruvate, high density lipoproteins [136,137], and all display no differences between controls and TPP conjugated NPs in toxicity and immunogenicity evaluations. Hence, these TPP-modified NP systems are promising for antigen delivery to mitochondria as well.

(ii) Mitochondria targeting sequence (MTS): Mitochondrial proteins are encoded by nuclear gene and further synthesized by cytoplasmic free ribosomes [138]. The translocation and intracellular sorting of these proteins to mitochondrial compartments depend on MTS [139]. Thus, MTS can be used as the targeting moiety. MTS commonly have a length of 20-40 amino acids and are located at the amino terminal of the precursor proteins. MTS form amphiphilic α-helical conformations with cationic amino acids on one side and hydrophobic amino acid on the opposing surface [140,141,142]. Table 6 summarizes MTS as mitochondria targeting ligands and their targets in the protein import machinery pathway.

## 4. Examples of Targeted NP-Based Vaccine

To demonstrate how mitochondria targeting NP-based vaccines can be developed, here we will provide possible modifications for immune modulation and vaccine development.

T lymphocytes with CD4 and CD8 surface proteins are referred to as CD4^+^ and CD8^+^ T cells, respectively. CD8^+^ T cells are cytotoxic when activated within the immune system, whereas CD4^+^ T cells are helpers in activating CD8^+^ T cells or humoral immune responses [161]. The activation of CD8^+^ T cells requires the presence of APCs. DCs, the most effective APCs, present antigens to B and T lymphocytes for initiating antigen-specific immune responses or immunological tolerance through antigen and costimulatory molecules [162,163]. T-cell antigen receptors recognize antigens by binding APC surface major histocompatibility complex (MHC) molecules, whereas CD8^+^ T cells are activated by DCs [164,165]. Thus, DCs are attractive for therapeutics that rely on immune mediation, such as vaccines, and can be used to target infection, cancer, and tumor immunity [165,166,167].

DCs play an important in role in shaping adaptive immune responses. When activated, immature DCs express increased levels of co-stimulatory molecules, consequently leading to potent adaptive immune response [168]. Activation of DCs occurs in response to inflammatory chemokines, such as MCP1, MI-3α. DCs are recruited to the inflammation site, where they mature into functional DCs to regulate antigen capturing, processing, and expression of co-stimulatory molecules for activating T cells [165,169,170]. Because DCs are the primary APCs and their activation and maturation is crucial in shaping an effective T-cell response against invading pathogens or cancer cells, approaches aimed to increase uptake, activation and efficiency in antigen presentation should improve vaccine efficacy. In this way, NP-based antigen delivery systems can be used to drive immature DCs towards maturation for immune response activation against a delivered antigen. 

Chong *et al.* [38] reported PLGA NPs for co-delivery of hepatitis B core antigen (HBcAg) and monophospholipid A (MPLA), a ligand for TLR, as therapeutic vaccines. A PLGA NP system was used to deliver antigen and Th1 promoting adjuvant to DCs to enhance immune response. The study demonstrated a synergistic effect, in regards to the PLGA NP co-delivery system, with anti-HBcAg IgG detection in sera and robust T cell proliferation response in mice *via* a booster dose. IFN-*γ* produced by T cells from the spleen and lymph nodes were 4- and 6-fold enhanced in mice immunized with HBcAg + MPLA-NPs than HBcAg NPs, respectively. Furthermore, no IL-4, a cytokine associated with cytotoxic immune response dampening, was found. Pulsing synthetic tumor peptides to DCs was demonstrated to elicit protective and therapeutic antitumor immunity [171]. A mitochondria targeting NP vaccine can be achieved by modifying this PLGA-NP carrier with a mitochondria targeting ligand, such as TPP.

A mechanism in which a vaccine can confer immune protection against disease using APCs is through triggering the selective or abundant expression or release of internal antigen in dysfunctional cells. Apoptotic cells, tumor lysates, and TAAs are all types of internal antigens that can be recognized by APCs. Rahma *et al.* [172] reported that pre-immature DCs pulsed with HPV16 E6 or E7 peptide derived from early genes E6 and E7 in high-risk HPV types 16 and 18 were well tolerated and able to induce specific immune responses in patients for therapeutic cervical cancer. Other vaccines are being developed against TAAs for vaccination of patients with various cancers. Rong *et al.* [173] developed a DC-based vaccine using MUC1-peptide, a TAA associated with late stage pancreatic cancer. In preliminary studies, IFN-*γ* and granzyme B, markers for cytotoxic immune response, were significantly enhanced in some patients. Phuphanich *et al.* [174] demonstrated the feasibility, safety, and bioactivity of TAA peptide for autologous vaccines by pulsing a class 1 peptide TAA expressed in patient’s glioma onto DCs. The TAA peptide pulsed DC vaccine administered intradermally into patients was nontoxic and led to elimination of CD133^+^ recurrent tumors cells. It is possible to target mitochondria of dysfunctional cells using NP-based system to trigger increased expression of internal antigens, especially TAAs, by inducing apoptosis (and generating apoptotic cells) for APC recognition and vaccines.

Antigen simulating activity by adjuvant has potential for synergistic effects resulting in stronger immune responses compared to antigen or delivery system alone. Tamayo *et al.* [175] reported a poly(anhydride) NP system for Th1 adjuvant immunoprophylaxis and immunotherapy. Studies demonstrated that poly(anhydride) NPs can act as agonists of various TLRs (e.g., TLR2, -4, and -5), to induce Th1-cytokine production (IFN-*γ*, IL-12) and trigger the expression of CD54 and CD86 co-stimulatory molecules after incubation with DCs. The *in vivo* studies suggested that NPs help elicit CD8^+^ T cell response. The co-administration of empty NPs with OVA showed induction of cytotoxic T cells specific for target cells. Furthermore, IFN-*γ* was detected in splenocytes from mice immunized with NPs and OVA. Aluminum-based adjuvants are licensed in vaccine with long record of safety without side effects of immune complex disorders [176]. α-Al_2_O_3_ NPs is reported to be a promising adjuvant in therapeutic cancer vaccines [177]. *In vitro* and *in vivo* studies demonstrated that the required antigen necessary to activate T cells was reduced by using α-Al_2_O_3_ NP adjuvant system. Notably, tumor growth was inhibited for more than 40 days and high levels of OVA-specific T cells were detected in mice immunized by α-Al_2_O_3_-OVA NPs.

**Figure 2:**
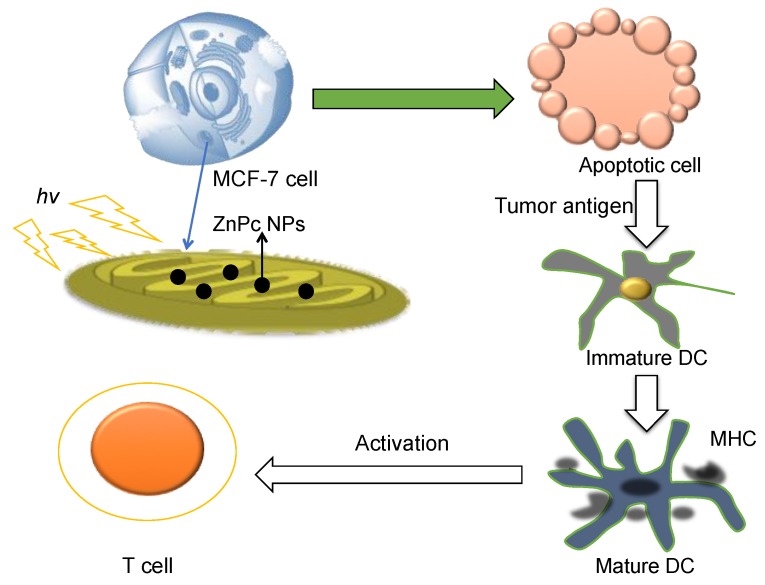
The schematic diagram of action of mitochondria targeted ZnPc NPs. Redrawn from Reference [21].

Photodynamic therapy (PDT) is a rapidly developing tactic for therapeutic vaccines. It has the advantage of accurately locating photosensitizer (PS) to the desired sites by light irradiation [178]. PS molecule can be excited to produce reactive oxygen species (ROS) [179,180]. The increase in ROS results in tumor cell apoptosis and stimulates the host’s immune response. A combination of PDT with mitochondria targeted NP is a possible strategy to stimulate the immune system. Our group [21] reported mitochondria targeted NPs containing ZnPc and combined with PDT to *ex vivo* stimulate DCs to secrete the cytokines, especially IFN-*γ* and DC mediated activation of CD8^+^ T cells by procuring antigens from MCF-7 breast cancer cells (Figure 2). The mitochondria-targeted polymer PLGA-*b*-PEG-TPP was employed as ZnPc delivery cargo with TPP cation as mitochondria targeting moiety. The targeted ZnPc NPs showed enhanced apoptotic properties compared to non-targeted NPs by triggering mitochondria-mediated apoptosis. The TAAs from apoptotic cancer cells were then released and internalized by DCs, which further led to the CD8^+^ T cell activation. Cytokines IL-18 and IFN-γ were secreted by bone marrow derived DCs stimulated by apoptotic cancer cells produced by mitochondria targeted ZnPc NP treatment in the presence of light.

These results indicated that mitochondria-targeted-NP delivery systems containing mitochondria-acting photosensitizers are suitable for activating tumor cells which can further activate DCs for subsequent immune response. Our group also reported the combination of ZnPc and CpG-ODN in PLGA-*b*-PEG polymer carrier, which resulted in significant phototoxicity of 4T1 metastatic mouse breast carcinoma cells [28]. The PLGA-*b*-PEG-NP system was modified with CpG-ODN-coated gold NPs on the surface. The CpG-ODN–Au–ZnPc–Poly-NPs were highly toxic to 4T1 cells under irradiation [28]. These results suggest that patients with light accessible cancers may be treated by administration of a PDT active mitochondrial targeting vaccine NP systems that capitalize on optimizing immune function for cancer cell death and prevention, overall preventing the need for harsh chemotherapeutics and decreasing the rate of recurrence *via* lingering immune surveillance by memory cells.

Besides DCs, microfold cells (M cells), specific epithelial cells of mucosa-related lymphoid tissues that transport antigens from the lumen to immune cells, and macrophages can also initiate immune response and/or tolerance. Thus, it is possible to target mitochondria of macrophages and/or M cells with mitochondria-targeted NPs for therapeutic vaccine [181]. Fievez *et al.* [182] reported NPs with non-peptide ligands for targeting M cells for oral vaccination. The targeted OVA NPs showed enhanced cellular immune response with high levels of IFN-*γ* production and consistently low levels of IL-4 in splenocytes. *In vivo* studies in mice indicated higher IgG immune response than non-targeted formulations. Chen *et al.* [183] reported liposomal NPs as antigen delivery systems for macrophages. By decorating liposomes with 3’-BPCNeuAc ligands and glycan ligand, delivery of the antigens to endosomes and lysosomes, respectively, were achieved. Results demonstrated that liposomal NPs were efficient in delivering OVA to bone marrow derived macrophages and significantly enhanced T cell proliferation. By modifying the liposome with mitochondria targeting moiety, it will be possible to target the antigen NPs into mitochondria for efficient antigen delivery and immune response. Zhou *et al.* [184] reported that graphene nanosheets of diameter 172.7±75.6 nm and of thickness 2–3 nm induced the secretion of Th1/Th2 cytokines (IL-1α, IL-6, IL-10, TNF-α) and chemokines (GM-CSF MCP-1, MIP-1α, MIP-1β, RANTES) in murine macrophages. These settings of NPs can be potentially designed to target mitochondria by conjugating or surface modified with mitochondria targeting moiety.

Most human tumors are MHC class II negative, which CD4^+^ T cells cannot recognize. NPs have potential to elicit the immunomodulatory cytokines which favor the proliferation and differentiation of cell-mediated immunity. Sena *et al.* [13] reported that increased mitochondrial ROS was able to produce nuclear factor of activated T cells and subsequent IL-2 cytokine expression. Specifically, the study demonstrated that mitochondrial complex III ROS is required for CD4^+^ T cell activation in mice model. The Uqcrfs1^−/−^ T cells, lacking mitochondrial complex III ROS and mitochondrial production of ATP, and controls WT CD4^+^ T cells were isolated from Cd4-cre mice, which had the same cell viability after 24 h of cell culture. The Uqcrfs1^−/−^ CD4^+^ T cells failed to induce IL-2 and were less activated (reduced CD69 and CD25 markers) in response to anti-CD3 and anti-CD28 stimulation. Hence, mitochondria targeting to immune cells (e.g., T cell, natural killer cells) by NP-systems can be a promising method to enhance cytokine generation efficiency *via* mitochondria pathways.

Some efforts were made towards using NPs to potentiate cytokine generation for immune response. For example, Hanley *et al.* [185] reported ZnO NPs as modulators of pro-inflammatory cytokines, IFN-*γ*, TNF-α, and IL-12 in primary human immune cells. ZnO NPs with size range of 4–20 nm were prepared by forced hydrolysis of zinc acetate. The 8-nm ZnO NPs were used to evaluate the generation of cytokines of immune cells in isolated peripheral blood mononuclear cells (PBMC). A pretreatment with low level of IFN-*γ* before treatment by ZnO NPs resulted in a significant amount of IL-12 expression, indicative of a synergistic relationship between ZnO NPs and IFN-*γ*. The ZnO NP treated PBMC showed significant enhancement of IL-12, IFN-*γ*, and TNF-α cytokine levels, which were dose dependent for ZnO NP. The cytokines (IFN-*γ*, TNF-α, and IL-12) produced by ZnO NPs aided in antigen processing and immune cell differentiation for enhanced destruction of cancerous cells, virally infected cells, and/or intracellular pathogens. ZnO NPs are also reported to enhance inflammatory cytokines levels in murine macrophages [186]. Liu *et al.* [187] reported that poly-hydroxylated metallofullerenol [Gd@C_82_(OH)_22_]_n_ modulates levels of Th1 (IL-2, IFN-*γ*, and TNF-*α*) and Th2 (IL-4, IL-5, and IL-6) cytokines in T cells and macrophages. The ratio of CD4^+^ to CD8^+^ T cells was significantly increased by [Gd@C_82_(OH)_22_]_n_ treatment. Polyvinylpyrrolidone (PVP)-PEG-Ag nanorods were reported for HIV vaccine [188]. Au NPs were modified with glycopolymer to be used as synthetic cancer vaccines [189] and showed strong antibody production. Further modification of these sets of NPs by mitochondria targeting moiety can potentially modulate immune response.

## 5. Conclusions and Future Outlook

Mitochondria play critical roles in cell life and death, and their dysfunction is indicative of mitochondria-associated diseases. An ever-increasing number of investigations are focused on mitochondria targeting for efficient disease therapeutics, indicating the potential for such strategy in preventive/therapeutic vaccine. Possible roles for targeting mitochondria in vaccine development can be: (1) as targets of APCs (e.g., DCs) for external antigen delivery systems; (2) as targeting sites of immune cells activation (e.g., T and B cell); (3) as targets in dysfunctional cells (e.g., tumor cells) for apoptosis and production of TAAs available for APC uptake and subsequent activation of immune cells. NP-based vaccine systems are promising for: (1) carriers for delivery of external antigens; (2) inducers of apoptosis for release of internal antigens; and/or (3) inducers of cytokine production *via* due to their intrinsic properties. There are relatively fewer studies concerning mitochondria-targeted NP vaccine delivery systems. Mitochondrial targeting NP systems can provide for both cell-mediated (Th1; CD8^+^ T cell) and humoral (Th2; B cell) immune response *via* activation of various pathways, such as complex III, ROS, and are promising tools for vaccines with effective immune responses. To achieve the mitochondria targeting purpose, the well-developed antigen nanocarriers, consisting of polymer-based and liposome-based antigen delivery systems, should be modified with a mitochondria targeting moiety, such as TPP or MTS, in order to direct antigen systems to the mitochondria. NP based vaccine delivery system has been shown to be a promising method for enhancing vaccine efficacies and can potentially be translated in to a worldwide immunization scope in the near future.

## Figures and Tables

**Table 1:** Summary of possible mitochondrial targets for vaccine development.

Cell type	Possible targets	Immune response	Possible Application	Ref.
Dendritic cell (DC)	Mitochondrial DNA (mtDNA)	Induces CD8^+^, IFN-*γ*, T cell response specific for tumor-associated mitochondrial antigens	Cancer	[19]
Cytolytic T lymphocytes	mtDNA	Controls the expression of maternally transmitted antigens	Hearing impairment	[23,24]
	Pyruvate dehydrogenase complexes	Increases CD8+ T cells for immune-pathogenesis of PBC.	Primary biliary cirrhosis	[25]
B cells	Mitochondrial permeability transition pore (MPTP)	Connects the B cell antigen receptor to the effector caspases of apoptotic cell death	acute cerebral ischemia	[26]
Breast cancer cell (MCF-7) and DCs	Mitochondrial matrix (MM)	Generates the apoptotic cancer cells providing tumor antigens for immune response	Cancer	[21,27]
4T1 cell	MM	Increases pro-inflammatory IL-2, IL-6, IL-12, TNF-*α* cytokines	Cancer	[28]
T cells	Bcl-xL/Bcl-2 proteins in outer mitochondrial membrane (OMM)	SARM causes T cell death by inhibiting Bcl-xL and down regulating signal-regulated kinase phosphorylation for immune homeostasis	Influenza	[29]
	2-oxo-dehydrogenase enzymes in inner mitochondria membrane (IMM)	Up regulates the expression of MHC class II, produces IL-2 cytokine in response to PDH-E2/BCKD-E2	Primary biliary cirrhosis	[30]
	Electron transport chain (ETC)	Generates ROS for the nuclear factor of activated T cells (NFAT) and IL-2 induction	Cancer	[31]
Cytolytic T lymphocytes	Pyruvate dehydrogenase complexes	Increases CD8+ T cells for immune-pathogenesis of PBC	Primary biliary cirrhosis	[25]

**Table 2:** Polymeric NP based antigen delivery systems.

Polymer System	Preparation/Diameter (nm)	Activity/Outcome	Delivery route	Comments	Ref.
PLGA	Double emulsion method/320 nm	OVA and MPLA dual loading PLGA NPs show enhanced mucosal immune response with higher IgA titers production than individually loaded NPs.	Oral	FDA approved delivery system, (OVA +MPLA) PLGA NPs were stable up to one month.	[50]
PLA	Dialysis method/300–600 nm	HIV-1 p24 PLA NPs show the best CTL results, antibody production, cytokine secretion (IL-2, 4, 6, 10, INF-*γ*) within the controls.	Subcutaneous injection	PLA NPs were stable for months	[51]
PGA	Dialysis method/200 nm	The hemagglutinin (HA) loaded PGA-NPs show enhanced CTL activity and greater production of IFN-*γ*, IL-4, and IL-6 *in vitro*. NPs vaccination shows better defense to influenza virus infection *in vivo* than controls.	Subcutaneous injection	Low cost, safe, relatively abundance, water-soluble, biodegradable	[52]
PMMA	Reflux-filtration methods	HIV-1 Tat Protein loaded PMMA NPs show efficient cellular uptake, well-patterned antigen release properties, and enhanced immune responses with greater proliferation index and cytokine level (INF-*γ*, IL-2) compared to Tat alone.	Intramuscular	Core-shell NPs were prepared. Tat was protected from oxidation. No severe damage was observed for Tat PMMA NPs.	[53]
PPS	Emulsion-incubation/size was not specified	OVA loaded PPS NPs with longer peptide showed greater cellular uptake, enhanced IFN-γ secretion, and T cell activation both *in vitro* and *in vivo*.	Tail vein injection	Surfactant pluronic F127 was used to stabilize NPs, PPS NPs internalized into cell via miscellaneous pathways.	[54]
PLA-PLGA	Double Emulsion-solvent evaporation method/450–800 nm	HBsAg co-polymeric NPs show increased immune responses with enhanced sIgA levels and greater production of cytokines (IL-2, IFN-*γ*) *in vivo*.	Intramuscular injection via pulmonary route	To deliver hepatitis B vaccine; Certain toxicity to pulmonary epithelium still exists. Limited for oral vaccine delivery	[55]
PLGA-PEG-TPP	Nano-precipitation method	ZnPc loaded co-polymeric NPs showed greatly enhanced T cell activation with combination of photodynamic therapy.	*Ex vivo*	Copolymer is of non-immunogenic and nontoxic, and designed for mitochondria targeting delivery.	[21,56]
PEG-PLA-PEG	Double emulsion & solvent evaporation/215 nm	The co-polymeric NPs showed elevated immune response *in vivo*. Cytokine levels (IFN-*γ* and IL-2) were greatly enhanced.	Oral	The NPs was stable in gastric and intestinal fluids. 90% of hepatitis B antigen was encapsulated.	[57]
PCL–PEG–PCL	Emulsion-solvent evaporation method/137 nm	The co-polymeric NPs delivery of bFGF antigen induces better antibody production for immune response *in vivo* than antigen alone.	Subcutaneous injection	A few studies have been made on this co-polymeric system.	[58]
Chitosan	Ionotropic gelation technique/160–200 nm	rHBsAg loaded chitosan NPs induced pretty delay immune response but much greater production of IgG than conventional alum vaccines *in vivo*.	Intramuscular or intranasal	NPs could be damaged by centrifugation-resuspension cycles. NPs could release antigen in a well-controlled pattern.	[46]
Chitoson-PLGA	Emulsification-solvent extraction/448 nm	Chitoson/PLGA NPs show gradual release of OVA up to 100% in 15 days, effective cellular uptake by crossing nasal epithelium, efficient T cell proliferation and stimulation *in vivo*.	Nasal	NPs charge, size, and antige release properties are critical factors for vaccination.	[59]

PLA: poly(d,l-lactide); PCL: poly(ε-caprolactone); PGA: poly(*γ*-glutamic acid); PLGA: poly(lactic-co-glycolic acid); PEG: Polyethylene glycol; PCL: poly(*ɛ*-caprolactone); PPS: Poly(propylene sulfide); PMMA: poly(methylmethacrylate).

**Table 3:** A summary of liposome-based antigen delivery systems.

Liposome Type	Example	Advantage	Disadvantage
Liposomal NP	E7 Peptide vaccinates against HPV [14]	No hypersensitivity reactions	Vulnerable to deoxyribonulease
	Plasmid DNA vaccinates against influenza [77]		
	HSP70 targets tumors [78]		
	D. pteronyssinus vaccination againsts asthma [79]	Do not create antibodies against the phospholipid components	Do not target antigen-presenting cells well
	DNA-hsp65 vaccinates against tuberculosis [80]	Can release antigens over long period of time	Short systemic half life
	Hepatitis A virus vaccinates against Hepatitis A [81]	Potential to cross epithelial barriers	Difficulty keeping certain molecules encapsulated
	HIV type 1 vaccinates against AIDS [82]	Low toxicity	
Solid Lipid NP	Cystosine-guanine containing oligodeoxynucleotides (CpG ODN) antigen treats allergies and inflammatory disease [83]	Stimulate a more effective immune response due to a good pharmacokinetic profile	Poor stability and biodistribution
		Capable of reversible denaturation	Low loading capacity
	Protein antigen vaccinates against hepatitis B and malaria [84]	Quick production time	Colloidal structures are present
Liposome-polycation-DNA (LPD)	HPV 16 E7 protein used to vaccinate against cervical cancer and HPV [85]	Safe toxicity profile	Most effective targeting is with proteins
		The plasmid DNA and cationic liposomes are immunostimulatory	
Polymerized Liposomes	Cationic antimicrobial peptides (AMPs) vaccinates against Pseudomonas aeruginosa [86]	Stable in the GI tract	Inconsistent targeting

**Table 4:** Summary of other NPs-based antigen delivery systems.

NPs	Example	Advantage	Disadvantage	Ref.
Surface-Modified Diamond NPs	Mussel Adhesive Protein (MAP) antigen	Strong and specific antibody response	Studies show that the NPs may adhere to the GI tract and block gut cells	[87,88]
		NPs have efficient surface exposure		
Gold NP	T-helper ovalbumin_323–339_ peptide (OVA_323–339_), CpG1668 oligodeoxynucleotide	Able to deliver fully synthetic carbohydrate-antigens, larger accumulation in a local lymph node	They are highly polarizable and are prone to aggregation	[89,90]
Silver NP	CD4 and gp120 for HIV and monkey pox	Exhibit antiviral tendencies	Tests show that these NPs aggregate in the presence of cations	[91,92]
		Has electrostatic double layer repulsion which stabilizes dispersion		
Aluminum Oxide NP	HIV gp120 C4 antigen for HIV	Less inhibited by pinocytosis and phagocytosis once in the body	Tend to aggregate when the pH changes	[93,94]
			Surface charge is not particularly stable	
Interbilayer-crosslinked multilamellar vesicles	VMP001- protein based malaria antigen for malaria	Elicit a powerful T-cell response	Rapid release when exposed to endolysosomal lipases	[95]
Papaya Mosaic Virus Capsid Protein NP (PapMV)	Nucleoprotein Antigen for influenza	Very stable NP	Only been used when working with influenza	[96,97]
Single Walled Carbon Nanotubes	Prostate-Specific Antigen for prostate cancer	High affinity for graphite structures	Poor survival times	[98]
		High selectivity		
		Active immune response		
Silica NP	Bovine Serum Albumin for HIV, influenza, and Hepatitis	Chemically stable, good biocompatibility, low toxicity	Ineffective for quick release	[99]
Calcium Phosphate Adjuvant	Mucosal delivery of herpes simplex virus type 2 antigen against the herpes virus	Very low toxicity	Tendency towards adverse reactions	[100,101,102]
	Epstein-Barr virus proteins against Epstein-Barr virus			
	Diphtheria Toxoid against Diphtheria	No detectable immunoglobulin E response	Relatively small binding capacity	
	Tetanus Toxoid against tetanus			
Aluminum Phosphate Adjuvant	Hepatitis B surface antigen against Hepatitis B	Enhance antibody responses in DNA vaccines	Thermal stability of the protein is reduced once absorbed	[103,104]
		Proteins absorb well if oppositely charged		
Virus-like Particles	HPV-16/18 against human papilloma virus	Can be produced for mucosal delivery	Incapable of co-expression	[105,106,107]
		Cheap production		
	Hepatitis B core antigen against Hepatitis B	VLP size is favorable for being taken up by dendritic cells	Not readily taken up by cells other than DCs	
Lipopeptides	Hepatitis B vaccine, Human immunodeficiency virus vaccine	Highly immunogenic	Require organic solvents or detergents	[108,109,110]
		Do not need ad adjuvant	Poor stability over time	
Bacterial DNA	Ovalbumin antigen against tumor growth	Activate natural killer cells	Low immunogenicity	[111,112,113]
	Gp140 against human immunodeficiency virus	Cost efficient	DNA is subject to degradation	
	Hepatitis B core antigen against Hepatitis B	Non toxic		
Lipopolysaccharide	*Brucella* against brucellosis	Biodegradable	High toxicity	[114,115]
	Allergy vaccines	Good binding	High inflammatory response	
Layered double hydroxide	Ovalbumin against tumor, DNA against melanoma	Low toxic, biocompatible, controllable antigen release	Toxic activity of LDHs still exists in *in vitro* and *in vivo* models	[75,116]

**Table 5:** Summary of the lipophilic cations as mitochondria targeting moiety.

Targeting moiety	Examples	Outcome	Ref.
Phospholipid (PL)-PEG-NH_2_	Single walled carbon nanotube functionalization (SWNT-PL-PEG)	To reduce nonspecific binding effect of SWNT surface. To improve the solubility of SWNTs in aqueous solutions. To accumulate in the mitochondria of normal and cancer cells	[121]
TPP^+^	PLGA-PEG-TPP as carrier for ZnPc	To induce cytotoxicity in cancer cells under light irradiation, which is used to activate DCs	[21]
Rhodamine 123	Liposomes-rhodamine-123-conjugated polymer	Least toxic among the liphophilic dye	[118,122]
		Facilitate the cellular association and internalization, direct the trafficking of NPs to mitochondria, and substantial cell killing was observed as the drug cargo	
Methyltriphenyl phosphonium	NA	Did not protect against cell death.	[119,123]
		Δ*ψ*_m_ was selectively depolarized	
Dequalinium (DQA)	DQA-PEG(5000)-DSPE	To cause cell death by inhibiting the mtDNA synthesis	[124,125,126]
	DQA-PEG(2000)-DSPE)		
MKT-007	NA	A mitochondria localized cationic dye, causes selective death of cancer cells	[127]
F16	F16 conjugated with 5-fluorouracil	F16 was used as a vehicle, selectively inhibits tumor cell proliferation and dissipates Δ*ψ*_m_	[128,129]
*N*-Heterocyclic Carbene (NHC)	Gold(I)-NHC Complex	Au(I)-NHC complexes toxic to breast cancer cell (MDA-MB-231, MDA-MB-468), but not to normal cells	[130]

**Table 6:** Summary of peptides with a mitochondria targeting sequence.

Name	Sequence	Targets	Comments	Ref.
Mitochondrial alanine aminotransferase (mALT)	MSATRMQLLSPRNVRLLSRGRSELFAGGSGGGPRVRSLISPPLSSSSPGRALSSVSATRRGLPKEKMTENGVSSRAKVLTIDT	Through interaction with translocases of the outer and inner mitochondrial membranes	Exhibits higher affinity for L-alanine	[143]
			Amino acids 1–83 contains MTS	
MTS-ExoIII-TAT-fusion protein	MLSRAVCGTSRQLAPALGYLGSRQ	Mitochondrial matrix	More efficient in mtDNA damage and less repair to cancer cell	[144]
AoPlaA	MLSCTSPLLRGACHNMGAAKALRLRWTVPPAVLIALGSGALYTTSGQTLYYKNSVQQTD	Mitochondrial intermembrane space	It is a cytosolic phospholipase A2 (cPLA2) like protein	[145]
p53 Protein	MLFNLRILLNNAAFRNGHNFMVRNFRCGQPLQ	Localizes within the membrane compartment	Mitochondrial accumulation of p53 is rapid, and precedes the apoptotic cascade.	[146]
SS peptide	2’,6’-dimethyltyrosine-D-Arg-Phe-Lys-NH_2_	Inner mitochondrial membrane	Prevents mitochondrial depolarization	[147,148]
	Phe-D-Arg-Phe-Lys-NH2			
	d-Arg-2’,6’-dimethyltyrosine-Lys-Phe-NH_2_			
XJB-5-131	4-hydroxy-2,2,6,6-tetramethyl piperidine-1-oxyl conjugated to nitroxide-Leu-D-Phe-Pro-Val-Orn	Mitochondrial membrane	ROS/RNS scavenger	[149]
Gramicidin S	Boc-Leu-^D^Phe-Val-Orn(Cbz)-OMe	Mitochondrial membrane	Electron scavenger	[150]
Nitroxide/Hemigramicidin S Conjugate	Hemigramicidin S-4-amino-2,2,6,6-tetramethyl-piperidine-N-oxyl (hemi-GS-TEMPO) 5-125	Accumulates at the interface of mitochondrial membrane	Acts as electron scavenger and provides the radioprotection of gamma	[151]
COX1_291–306_	MFTVGLDVDTRTYFT	mtDNA	Stimulates the CD8^+^ IFN-*γ*^+^ T cell response specific for tumor-associated mitochondrial Ags	[19]
Cytochrome P450 2E1 (P450 MT5)	MAVLGITVALLGWMVILLFI	Mitochondrial out and inner membrane	Reacts with cytochrome P450 in mitochondria	[152]
Activating transcription factor associated with stress-1 (ATFS-1)	AAVAYREAARAE	Inner mitochondrial membrane	ATFS-1 is degraded in mitochondria, which helps to maintain the mitochondrial homeostasis	[153]
KLA peptide	_D_(KLAKLAK)_2_	Mitochondrial membrane	KLA lysine units interact with the membranes for mitochondria uptake via hydrogen bonding and electrostatic attraction	[154]
RLA peptide	_D_[RLARLAR]_2_	Mitochondrial outer membrane	The substitution of D-lysines in KLA with D-arginines improves the plasma membrane permeability and increases mitochondrial accumulation of RLA (as early as 6 min)	[155]
Mitochondrial open reading frame of the 12S rRNA-c (MOTS-c)	MRWQEMGYIFYPRKLR	mtDNA	16-amino-acid peptide, which promotes metabolic homeostasis and prevents the obesity and insulin resistance	[156]
Y- or M-conjugate	NH_2_-MLSLRQSIRFFKPAT-o-o-N-TTCCTCGCTCACT-c (Y conjugate)	Matrix	Accesses into the matrix through outer and inner mitochondria protein import channels	[157]
	NH-MALLRGVFIVAAKRTPF-o-o-_N-_GATTCTTCACCGT_-C_ (M-conjugate)			
Mitochondria-penetrating peptides (MPPs)	F_X-_r-F_X-_K_-_F_X-_r_-_F_X-_K, F-r-F-K-F-r-F-K, F-r-F_X_-K-F-r-F_X_-K, F-r-Y-K-F-r-Y-K, F_X_-r-F_X_-K,F-r-F-K, F-r-F_X_-K, F-r-F_2_-K, F-r-Nap-K, F-r-Hex-K, F-r-Y_Me_-K, F-r-F_F_-K, F-r-Y-K, Y-r-Y-K	Matrix	Systematic series of MPPs were studied, delivery of nonpolar species into mitochondria has been demonstrated to be successful	[158]
MTS-Cys peptide	NH_2_-MVSGSSGLAAARLLSRTFLLQQNGIRHGSYC	Mitochondrial outer membrane	MTS peptide can be enhanced slightly outer stearyl-R8 modification	[159,160]
